# Dissecting efficiency of a 5’ rapid amplification of cDNA ends (5’-RACE) approach for profiling T-cell receptor beta repertoire

**DOI:** 10.1371/journal.pone.0236366

**Published:** 2020-07-23

**Authors:** Yu-Hung Lin, Sheng-Jou Hung, Yi-Lin Chen, Cheng-Han Lin, Te-Fang Kung, Yi-Chun Yeh, Joseph T. Tseng, Tsunglin Liu

**Affiliations:** 1 Department of Biotechnology and Bioindustry Sciences, National Cheng Kung University, Tainan, Taiwan; 2 Molecular Diagnostic Laboratory, Department of Pathology, National Cheng Kung University Hospital, Tainan, Taiwan; University of California San Francisco, UNITED STATES

## Abstract

Deep sequencing of T-cell receptor (TCR) genes is powerful at profiling immune repertoire. To prepare a TCR sequencing library, multiplex polymerase chain reaction (mPCR) is widely applied and is highly efficient. That is, most mPCR products contain the region critical for antigen recognition, which also indicates regular V(D)J recombination. Multiplex PCR, however, may suffer from primer bias. A promising alternative is 5’-RACE, which avoids primer bias by applying only one primer pair. In 5’-RACE data, however, non-regular V(D)J recombination (e.g., TCR sequences without a V gene segment) has been observed and the frequency varies (30–80%) between studies. This suggests that the cause of or how to reduce non-regular TCR sequences is not yet well known by the science community. Although it is possible to speculate the cause by comparing the 5’-RACE protocols, careful experimental confirmation is needed and such a systematic study is still not available. Here, we examined the 5’-RACE protocol of a commercial kit and demonstrated how a modification increased the fraction of regular TCR-β sequences to >85%. We also found a strong linear correlation between the fraction of short DNA fragments and the percentage of non-regular TCR-β sequences, indicating that the presence of short DNA fragments in the library was the main cause of non-regular TCR-β sequences. Therefore, thorough removal of short DNA fragments from a 5’-RACE library is the key to high data efficiency. We highly recommend conducting a fragment length analysis before sequencing, and the fraction of short DNA fragments can be used to estimate the percentage of non-regular TCR sequences. As deep sequencing of TCR genes is still relatively expensive, good quality control should be valuable.

## Introduction

In the adaptive immune system, T cells recognize a wide variety of antigens via expressing numerous distinct T-cell receptor (TCR) proteins. The diversity of a TCR gene stems from the plenty of exons that can be classified into variable (V), diverse (D), joining (J), and constant (C) gene segments. For example, the human TCR-α gene contains 54 V and 61 J gene segments while the TCR-β gene contains 67 V, two D, and 13 J gene segments [1]. During V(D)J recombination, one of each V, D (for TCR-β and TCR-δ), and J gene segments are selected and concatenated at the DNA level. In addition, random nucleotide deletion and insertion occur within the complementarity determining region 3 (CDR3), which is critical for antigen binding. These processes give rise to a huge number of distinct recombined TCR genes and the collection of CDR3 sequences (or clones) is often used to characterize immune repertoire.

The development of high-throughput next-generation sequencing (NGS) has enabled a comprehensive detection of diverse recombined TCR genes [2]. To prepare an NGS library of TCR genes, a widely applied approach is multiplex PCR, in which multiple primers are designed to bind all V and J or C gene segments for amplification [3]. This is highly efficient because most (>90%) mPCR products will contain both the V and J gene segments (defined as regular here). Multiplex PCR, however, likely suffers from primer bias, which can distort the resulting TCR repertoire [4]. Although modifications have been proposed to reduce the primer bias of multiplex PCR [5], complete removal of bias is still not warranted. The un-biased 5’-RACE is a promising alternative for preparing a TCR library as it amplifies TCR genes using only one primer that targets the constant region and a universal primer concatenated to the 5’ end [6]. Note that multiplex PCR can take both genomic DNA and RNA as input while 5’-RACE can be applied only on RNA samples. The selection of starting material is study-dependent [7]. As RNA provides information on gene expression, it better reflects the immune repertoire at the functional level.

Although a 5’-RACE approach avoids primer bias, it may not be efficient in rendering regularly recombined TCR sequences. For example, Fang et al. conducted deep sequencing of TCR-β transcripts amplified from lymphocytes in peripheral blood of non-small cell lung carcinoma (NSCLC) patients and found that on average only 19% of the TCR-β sequences were regular while 61% contained only the (D)J-C but not V gene segments [8]. In those non-regular sequences, most of the (D)J-C segments were preceded by introns at the immediate upstream, suggesting in-complete V(D)J recombination and/or abnormal splicing. Non-regular recombination in 5’-RACE data has also been reported in other studies. In a survey of TCR-β repertoire in metastatic melanoma tissues of ten patients undergoing cancer immunotherapy [9], the authors showed a large variation (8–75%) in percentage of regular reads. On average, the fraction of effective reads containing a CDR3 clone was only 40%. In another study, TCR-β repertoire in peripheral blood and renal graft biopsies of kidney transplant recipients were examined, and only 38% and 33% of the reads contained a CDR3 clone for the two types of samples respectively [10]. In contrast, some studies showed an efficient 5’-RACE sequencing of immune receptor genes. For example, Fang et al. reported an 81% regular TCR-β sequences for a healthy individual in their study of NSCLC [8].

The variation in fraction of regular data may be explained by several factors, e.g., sample source and quality, the specific immune gene under survey, and the diversified 5’-RACE protocols. Ruggiero et al. proposed a ligation-anchored magnetically-captured PCR method, which combines the 5’-RACE and a target enrichment approach, to investigate TCR-α and TCR-β repertoire [11]. They found that introducing magnetic capture increased the percentage of regular data from 37% to 70% on average. This indicates the importance of protocol in data efficiency. However, magnetic capture does not fully explain the variation because a 5’-RACE protocol without magnetic capture could also achieve a high fraction of regular reads [8]. It is possible to dissect the variation in data efficiency by comparing the 5’-RACE protocols. However, a cross-study comparison is difficult when the sample sources and/or target genes under survey are different. In addition, 5’-RACE involves many steps, which implies a large parameter space to be explored. Sample quality and labor operation could also affect the data efficiency. Therefore, a systematic investigation on the efficiency of 5’-RACE data is important and such a study is still not available.

Here, we searched for factors that affect the percentage of regular TCR-β reads in 5’-RACE data. To control for variation between protocols, a commercial kit was selected and tested on a control RNA and several peripheral blood samples. We found that the presence of short DNA fragments in the 5’-RACE library was the main cause of non-regular TCR-β reads in the data. We also demonstrated that filtering short DNA fragments in two steps significantly increased the fraction of regular data. Although two-step filtering has been applied in some studies [8, 12], our systematic investigation justifies the logic behind. More importantly, the discovery allows experimentalists to assess the quality of a 5’-RACE library before the expensive sequencing. The 5’-RACE protocol can also be tuned to optimize data efficiency based on the experimental metric of short DNA fragments.

## Materials and methods

### Blood sample preparation

This study was approved by the Clinical Trial and Research Ethical Committee, National Cheng Kung University Hospital (IRB No. A-ER-107-331), and informed written consent was obtained from each participant. With the informed consent, blood samples were obtained from two healthy Asian donors (L: male, age 44; K: female, age 32). Peripheral blood mononuclear cells (PBMCs) were isolated immediately from each sample by centrifugation. Total RNA of PBMCs was extracted using Trizol (Thermo Fisher Scientific Inc., USA) according to the instruction manual. The concentration and integrity of total RNA were determined on the Qubit Fluorometer (Thermo Fisher Scientific Inc., USA) and fragment analyzer (Agilent, USA).

### 5’-RACE protocol

Our 5’-RACE was performed using the SMARTer Human TCR-a/b Profiling kit (Takara Bio Inc., Japan). According to the manufacturer’s instructions, first-strand cDNA was synthesized using TCR dT primer (1.2 μM) and SMART-Seq Oligonucleotide (2.4 μM) in a 20 μL reaction volume, which contained 1 μg of total RNA, 1× first-strand PCR buffer, RNAase inhibitor (1U) and reverse transcriptase (10U). PCR extension was performed by a thermal cycler at 42°C for 45 min, followed by inactivation at 70°C for 10 min. For semi-nested PCR, the first-PCR run selectively amplified the total first-strand cDNA by SMART primer (0.12 μM) and TCR-b human primer-1 (0.12 μM) in a 50 μL reaction volume, which contained 1× PCR Buffer and DNA polymerase. A 1 min denaturation at 95°C was followed by 21 cycles of 1 min at 95°C, 1 min at 53°C and 1 min at 68°C, as well as a final extension at 72°C for 10 min. In the second-PCR run, 1 μL of PCR products from the fist-PCR run were amplified by TCR-forward primer (0.12 μM) and TCR-b human primer-2 (0.12 μM) in a 50 μL reaction volume, which contained 1× PCR buffer and DNA polymerase. A 1 min denaturation at 95°C was followed by 18 cycles of 1 min at 95°C, 1 min at 53°C and 1 min at 68°C, as well as a final extension at 72°C for 10 min.

### Purification of amplified libraries and high throughput sequencing

For size selection, PCR products were isolated at approximately 700 bp by AMPure XP (Beckman Coulter Inc., USA) and/or polyacrylamide gel. Three independent methods were performed: AMPure alone (A), gel extraction alone (G), and AMPure with gel extraction (AG). In method A, 25 μL of AMPure XP was added directly to products from the second-PCR run and the supernatant was transferred to a clean tube. The supernatant was incubated with 10 μL of AMPure XP for 5 min and removed after incubation. The cDNA Library bound to the AMPure XP beads was re-suspended in a 20 μL volume. Note that the original 5’-RACE protocol of the SMARTer kit applies AMPure filtering in two steps for filtering relatively long and short DNA fragments and was applied to the control RNA sample. We later reasoned that only the filtering of short DNA fragments mattered and retained only the corresponding AMPure step for preparing all the rest libraries. In method G, 20 μL products from the second-PCR run were directly run on a 4% polyacrylamide gel at 120V for 120 min. The fraction at approximately 700 bp was excised and purified. In method AG, products from the second-PCR run were directly incubated with 35 uL AMPure XP for 5 min, after which the supernatant was removed. The library bound to the AMPure XP beads was re-suspended in a 20 μL volume. The library was then processed using method G. Libraries with a fragment length of ~700 bp were validated with a fragment analyzer (Agilent, USA). Except for the control sample, all libraries were pooled together for one run of Illumina MiSeq 2×300 bp sequencing following the manufacturer’s instructions. Sequences were exported from the fluorescent images according to the Illumina data processing pipeline. The NGS data in this work are available in NCBI Sequence Read Archive (BioProject ID: PRJNA610460).

### Sequence analysis

Illumina raw paired-end (PE) reads were first merged into single reads using USEARCH (v11.0.667; command fastq_mergepairs) allowing a 25% mismatch rate within the overlap. Merged reads were analyzed using TRIg v1.0 [13], which annotated V(D)J recombination. Reads containing both V and J segments in a strict order were considered as regular and non-regular otherwise. Unmergeable PE reads were analyzed by TRIg separately. The resulting V(D)J annotations of first (R1) and second (R2) reads were then combined. In our experimental design, R1 should start with a constant segment, followed by a J segment, and usually extend into a V region. Therefore, an unmergeable PE was considered as non-regular if the R1 did not start with a C and J annotation or not followed by a V annotation. R2 was expected to span a large V region if the DNA fragment was regular. When R1 and R2 had identical V annotation, the V annotation was assigned to the pair. In some cases the V annotation of R1 was ambiguous (e.g., V6-2 or V6-3) because the V segment on R1 was too short to distinguish the two V genes. The V annotation of R2 can then be used to make certain the V gene if it was one of the ambiguous V annotations of R1. If R1 did not have a V annotation, the V annotation of R2 was used if available. Note that many V genes have two exons and for those V annotations we required that the J segment was connected to the second V exon followed by the first V exon if available. An unmergeable PE was considered as non-regular if the annotations of R1 and R2 were not consistent. For example, R2 had a V annotation different from that of R1, or R2 did not have a V annotation. In some cases, R2 had two different V or a non-V annotation (e.g., intergenic region). This suggested chimera and the PE was considered as non-regular.

To obtain CDR3 clones, we extracted regular data based on the TRIg annotations and used MiXCR v2.1.9 [14] to annotate the CDR3 region and corrected sequencing errors in the CDR3 clones. Because both merged reads and unmergeable PEs could be regular, we concatenated the regular merged reads and R1s of the regular unmergeable PEs as input for MiXCR. R1s of unmergeable PEs were used because they usually contained a full CDR3 segment. Note that reads with an ambiguous V annotation were excluded from the analysis. MiXCR output CDR3 segments with the flanking V and J annotations. However, some V-J annotations of MiXCR were different from those by TRIg. Therefore, we used the V-J annotations of TRIg and CDR3 segments of MiXCR to represent CDR3 clones after error correction. The corrected CDR3 clones were used to analyze reproducibility and obtain a saturation curve. For analysis of V-J composition, the data before error correction by MiXCR were used because MiXCR was more conservative and did not annotate some reads.

Linear regression, the statistics, and principal component analysis were done using the python (v3.7) package Scikit-Learn, and the results were plotted using the matplotlib package in python.

## Results

### Non-regular V(D)J recombination in 5’-RACE data

To confirm the presence of non-regular V(D)J recombination in 5’-RACE data, we applied the SMARTer 5’-RACE protocol to amplify human TCR-β genes from the control RNA sample in the kit. The control library C-A was then subjected to MiSeq 2×250 bp sequencing, which generated 96,496 PE reads (Table 1). Of the PE data, 38.7% could be merged into single reads for V(D)J annotation using TRIg. For unmergeable PEs, we aligned the first and second reads to the human TCR-β gene separately and found a small gap (<100 bp) between paired reads in most cases (S1 Fig). This indicated that most of the PEs could not be merged because the paired reads were not long enough to cover the whole DNA fragments, which were longer than 500 bp. The separate alignments of PE reads allowed us to combine the V(D)J annotations for judging the regularity of recombination. Putting together the merged reads and unmergeable PEs, 60.5% of the TCR-β sequences were found regular, i.e., composed of V-(D)J-C gene segments. Consistent with the previous study, a majority (63.1%) of the non-regular reads contained only the (D)J-C but not V gene segments. This confirms the presence of non-regular V(D)J recombination in 5’-RACE data.

**Table 1:** Statistics of sequencing data for all libraries.

Library	No. of raw PEs	No. (%) of mergeable PEs	No. (%) of regular sequences	No. (%) of corrected CDR3 clones	% of short DNA fragments
C-A	96496	37300 (38.7%)	58409 (60.5%)	54915 (94.0%)	5.2
L1-A	3083953	2913875 (94.5%)	963896 (31.3%)	879948 (91.3%)	16.5
L2-A	2554570	2426376 (95.0%)	733248 (28.7%)	659607 (90.0%)	18.6
L1-AG	1460605	1363539 (93.4%)	1240721 (84.9%)	1149563 (92.7%)	2.7
L2-AG	1378689	1273852 (92.4%)	1174941 (85.2%)	1081868 (92.1%)	4.5
K1-A	1949024	1778898 (91.3%)	957574 (49.1%)	833107 (87.0%)	9.4
K2-A	3368416	3105697 (92.2%)	1492075 (44.3%)	1305756 (87.5%)	13.7
K1-AG	1675636	1533437 (91.5%)	1554608 (92.8%)	1403029 (90.2%)	0.5
K2-AG	1547638	1409465 (91.1%)	1423839 (92.0%)	1264172 (88.8%)	0.6
K1-G	1526312	1434349 (94.0%)	1208264 (79.2%)	1077133 (89.1%)	4.2
K2-G	1509653	1427724 (94.6%)	1135241 (75.2%)	999473 (88.0%)	5.5

The percentages of mergeable PEs and regular sequences are relative to number of raw PEs. The percentage of CDR3 clones is relative to number of regular sequences. The percentage of short DNA fragments corresponds to the fraction of 250–600 bp fragments in the length analysis.

### Modified protocol for increasing fraction of regular sequences

**Fig 1:**
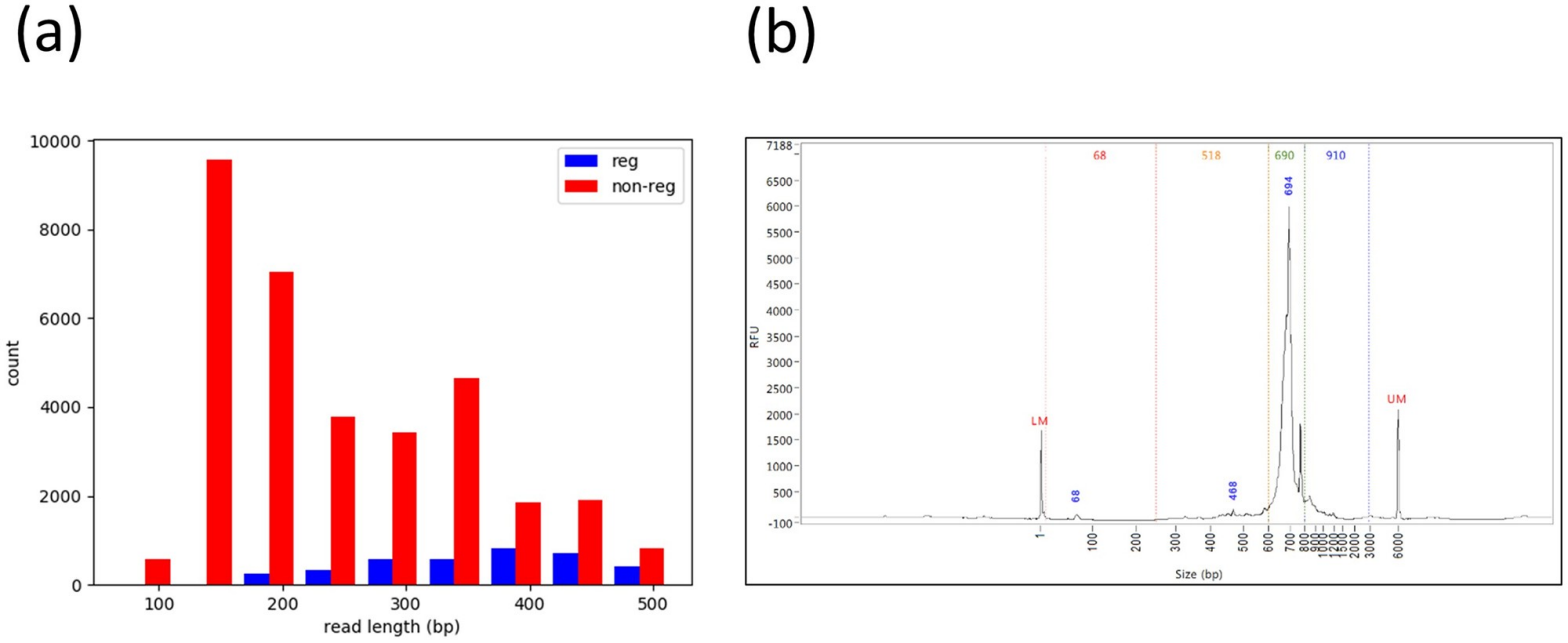
Length distribution of 5’-RACE products based on (a) the merged reads and (b) fragment length analysis of the C-A library. In Fig 1a, blue and red indicate regular and non-regular TCR-β sequences respectively.

Among the 37,300 merged reads, most (90.2%) were non-regular and the corresponding length distribution revealed that most (90.6%) non-regular reads were shorter than 400 bp (Fig 1a). On the contrary, most (92.5%) of the 59,196 unmergeable PEs were regular and the corresponding fragments should be longer than 500 bp. In other words, the majority of regular reads were longer than 500 bp, while the majority of non-regular reads were shorter than 400 bp. This motivated us to increase the fraction of regular sequences via filtering short DNA fragments before sequencing. Note that the SMARTer protocol already contains a step of filtering short DNA fragments using AMPure. We thus hypothesized that the AMPure step was not efficient enough to remove all short DNA fragments. In fragment length analysis, the DNA fragments contained adapter sequences and the fragments of size 250–600 bp were considered as short (Fig 1b). We set the lower limit as 250 bp because almost no DNA was observed below the limit except for a small peak likely contributed by primer dimers or other technical sequences. The upper limit was selected before the rise of the high peak, most of which likely represented regular TCR-β fragments. Indeed, fragment length analysis revealed a 5.2% short DNA fragments in the 5’-RACE products (Fig 1b and Table 1). Toward a complete removal of short DNA fragments, we proposed an additional gel extraction on the 5’-RACE products before sequencing. In the following, the original SMARTer protocol using AMPure and the modified one with an additional gel extraction were abbreviated as protocol A and AG respectively.

### Benefit of the additional gel extraction

**Fig 2:**
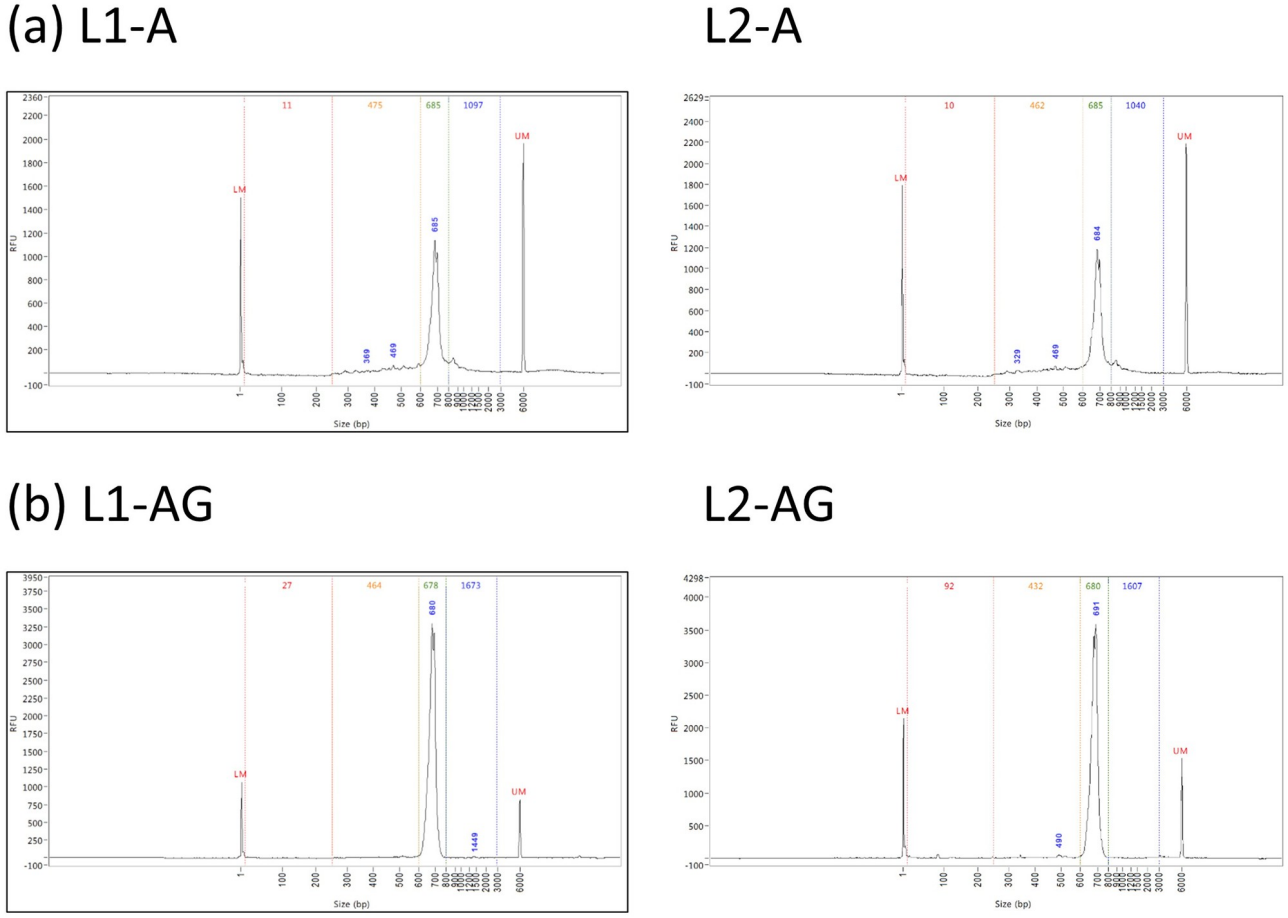
Fragment length distributions of 5’-RACE libraries constructed using protocol (a) A and (b) AG from blood sample of individual L. The two columns (L1 and L2) show results of the two technical repeats respectively.

To evaluate the additional gel extraction in raising the fraction of regular data, we prepared two 5’-RACE libraries, L1-A and L1-AG, from peripheral blood of a healthy individual L using protocols A and AG respectively (S2 Fig). Fragment length analysis showed a decrease in the fraction of short DNA fragments from 16.5% to 2.7% (Fig 2 and Table 1) with the additional gel extraction. This indicates the effectiveness of protocol AG in removing short DNA fragments.

The L1-A and L1-AG libraries were then subjected to MiSeq 2×300 bp sequencing, which generated 3,083,953 and 1,460,605 PEs respectively (Table 1). With the longer 300 bp reads, 94.5 and 93.4% of the PE reads could be merged into single reads. Again, the unmergeable PE reads were analyzed separately and the results were combined. Consistent with the fragment length analysis, the fraction of regular TCR-β sequences increased from 31.3% to 84.9% (Table 1) with the additional gel extraction. This validates the efficacy of protocol AG in producing regular data for studying immune repertoire.

To investigate the technical variation of the protocols, we repeated the above library preparations from the same blood sample for sequencing and analysis. For the repeat libraries L2-A and L2-AG, the additional gel extraction decreased the fraction of short DNA fragments from 18.6% to 4.5% and increased the fraction of regular TCR-β sequences from 28.7% to 85.2% (Fig 2 and Table 1). Compared to the first run, these similar numbers indicates the stability of the protocols.

**Fig 3:**
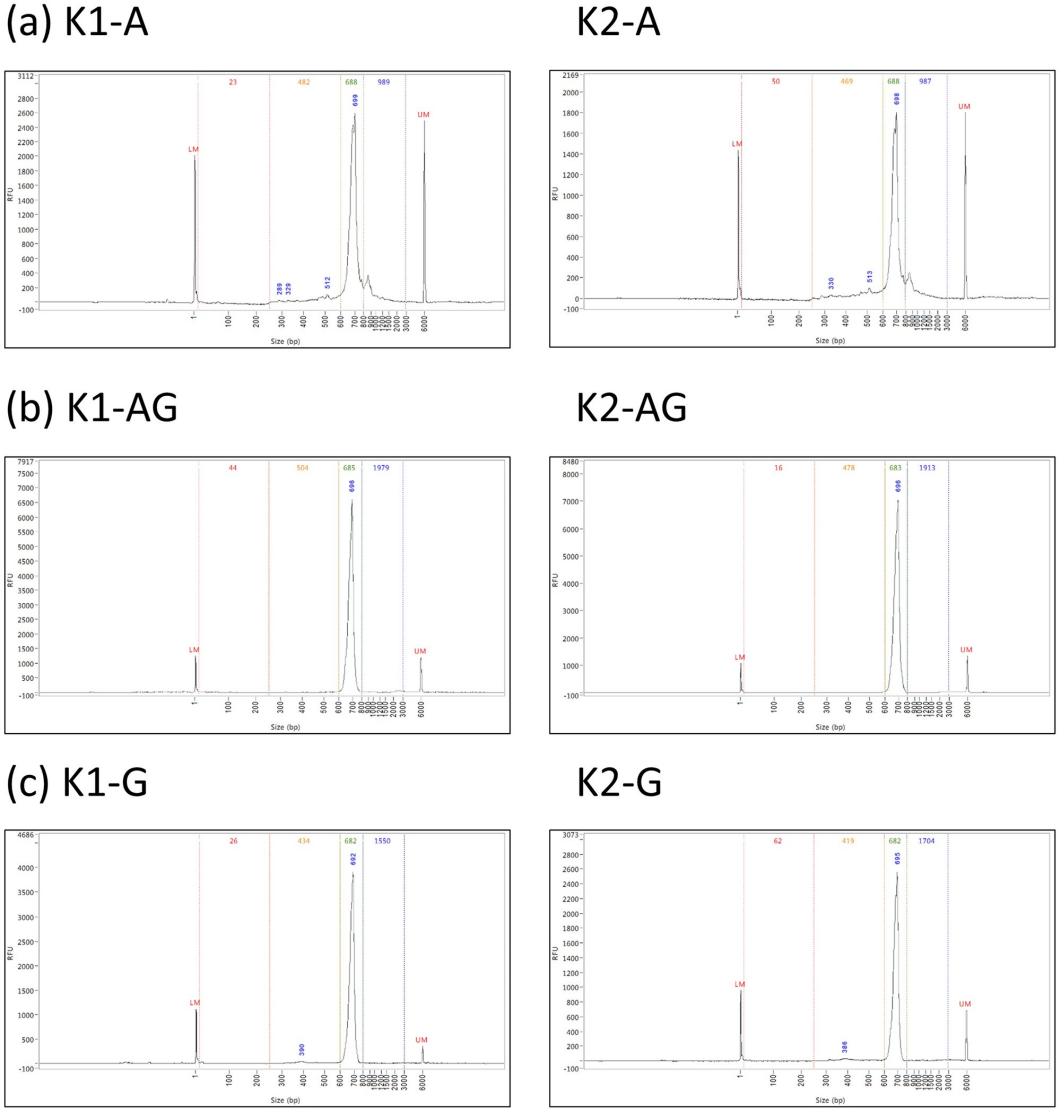
Fragment length distributions of 5’-RACE libraries constructed using protocol (a) A, (b) AG, and (c) G from blood sample of individual K with two technical repeats (K1 and K2) for each protocol.

To examine the generality of our findings, we repeated the above comparisons using blood samples of another healthy individual K (S3 Fig). Similar to the results of individual L, the additional gel extraction decreased the fraction of short DNA fragments from 9.4% to 0.5% and 13.7% to 0.6% in the two technical repeats respectively (Fig 3 and Table 1). Consistently, the fraction of regular TCR-β sequences increased from 49.1% to 92.8% and 44.3% to 92.0% in the two repeats. This confirms the general efficacy of protocol AG in producing regular TCR sequences.

The efficacy of protocol AG motivated us to examine whether a protocol using gel extraction only without the preceding AMPure step (called protocol G) could remove short DNA fragments to the same degree. Fig 3 and Table 1 show that protocol G could not remove short DNA fragments as efficiently as protocol AG. This suggests that the AMPure step before gel extraction could enhance the removal of short DNA fragments. Thus, to achieve a high fraction of regular TCR sequences, protocol AG is recommended.

### Estimating fraction of regular TCR sequences

Although protocol AG yielded a high (>80%) fraction of regular TCR-β sequences, we observed a variation in fraction among different sample sources (e.g., 84.9% and 92.8% for the L1-AG and K1-AG libraries respectively). The variation was even greater for protocol A (ranging from 28.7% to 60.5%). Those variations might be explained by the different sample natures and/or protocol implementations. Whatever the cause, this raises a concern that the yield of regular TCR-β sequences may fluctuate to an unsatisfying degree. It is thus helpful to keep track of the variation for enhancing protocol consistency.

**Fig 4:**
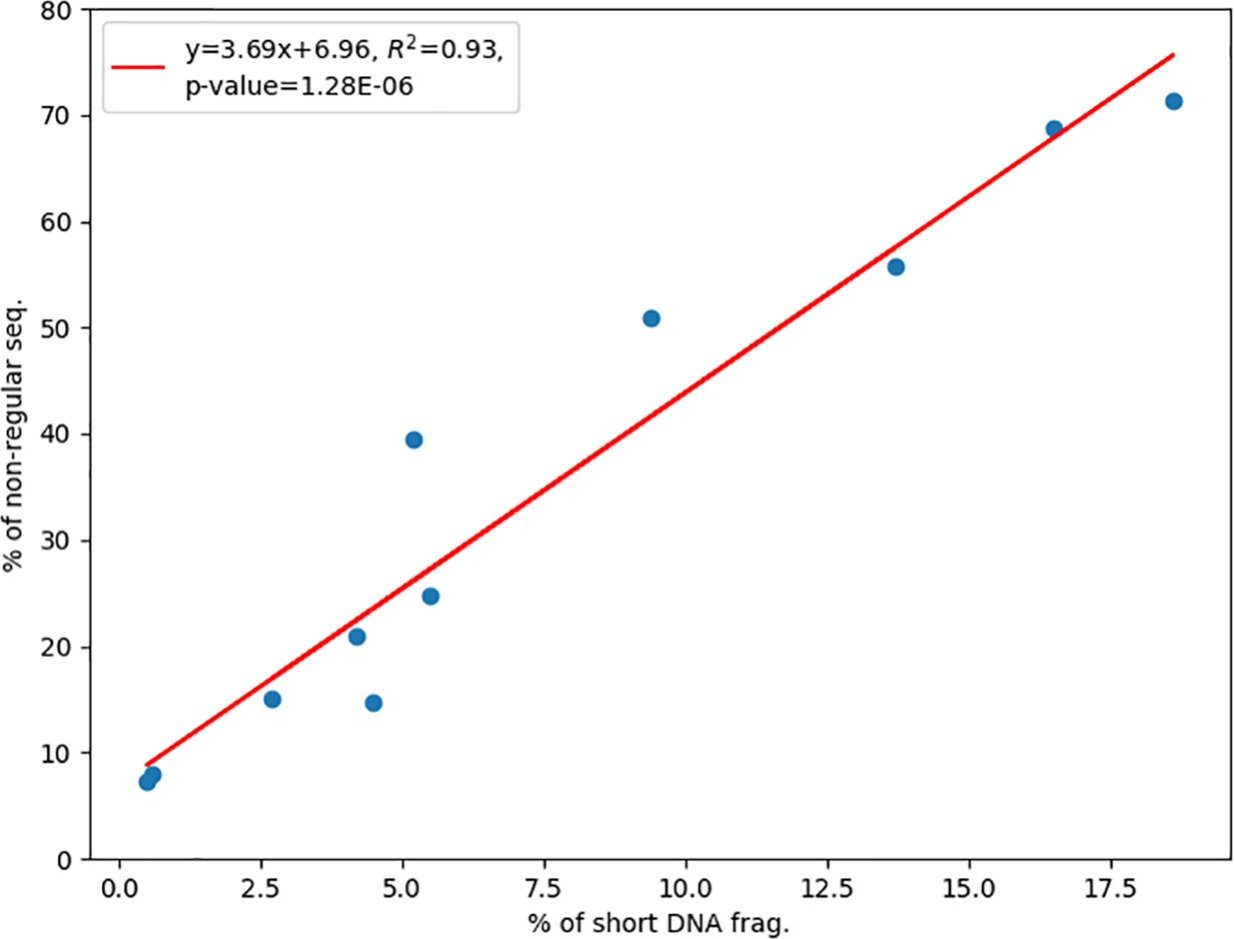
Correlation between the fraction of non-regular TCR-β sequences in the 5’-RACE data and the fraction of short DNA fragments in the library.

A plausible source of variation is the fluctuating efficiency of filtering short DNA fragments. Indeed, we found a strong linear correlation (R^2^ = 0.93) between the fraction of short DNA fragments and the fraction of non-regular TCR-β sequences (Fig 4). The strong correlation suggests that the source of variation could be largely attributed to the fraction of short DNA fragments. Although the fraction of short DNA fragments still can vary for different samples and/or protocol implementations, this metric is useful for controlling the yield of regular data and protocol optimization.

Although non-regular TCR-β sequences could be attributed to short DNA fragments, the cause of short DNA fragments in the 5’-RACE libraries was still not clear. We suspected that the quality of the RNA samples might contribute to the presence of short DNA fragments. To test this speculation, we measured the RNA integrity of the three RNA samples (S4 Fig). The corresponding integrity values were then compared to the fractions of short DNA fragments in the 5’-RACE libraries prepared by the same protocol A (Table 1). The control sample had the lowest RNA integrity, however, it did not show the highest fraction of short DNA fragments, which instead was observed in the 5’-RACE libraries of individual L. Therefore, RNA integrity could not explain the cause of short DNA fragments in the 5’-RACE libraries.

### Reproducibility of probed immune repertoire

**Fig 5:**
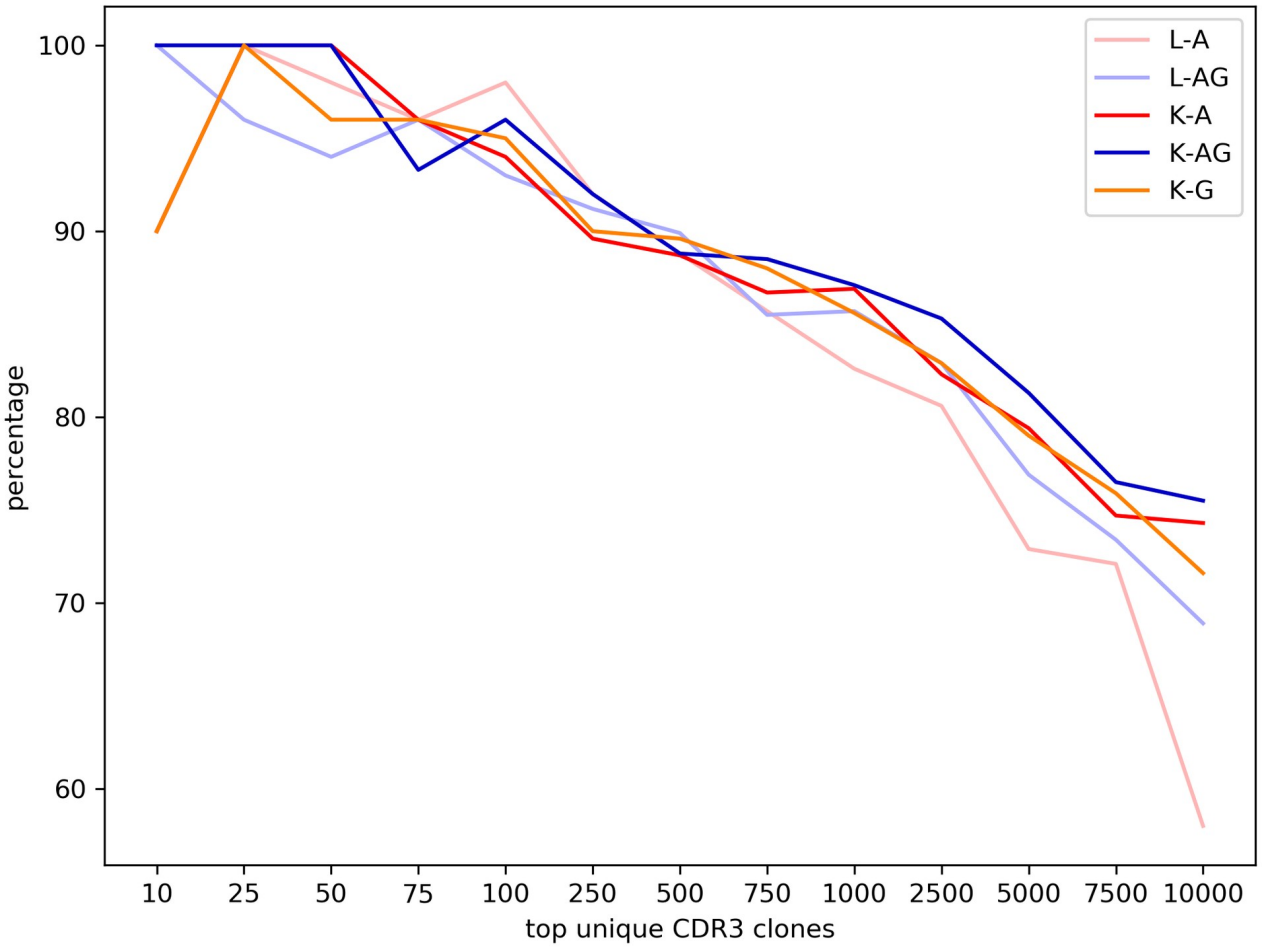
Percentage of common top unique CDR3 clones between two repeats of the five sets of 5’-RACE libraries.

The above repeats for assessing technical variation also allowed us to evaluate reproducibility of the protocols. To quantify reproducibility, we examined CDR3 clones within the regular TCR-β sequences and used MiXCR to correct sequencing errors in the CDR3 clones. The error-corrected CDR3 clones in the two repeats were then compared. For each set of CDR3 data, the abundance of unique CDR3 clones was calculated and the unique CDR3 clones were sorted by abundance from high to low. Among the two sets of top unique CDR3 clones, we counted the clones that appeared in both sets and the fraction was used to define reproducibility. Fig 5 shows that the reproducibility was similar for all protocols. Among the top 100 unique CDR3 clones, 93–96% were identical in the two repeats. When the top 1,000 and 10,000 unique CDR3 clones were examined, still ~85% and ~70% of the clones were identical in the two repeats respectively except for the L-A libraries.

### Immune repertoire by different protocols

**Fig 6:**
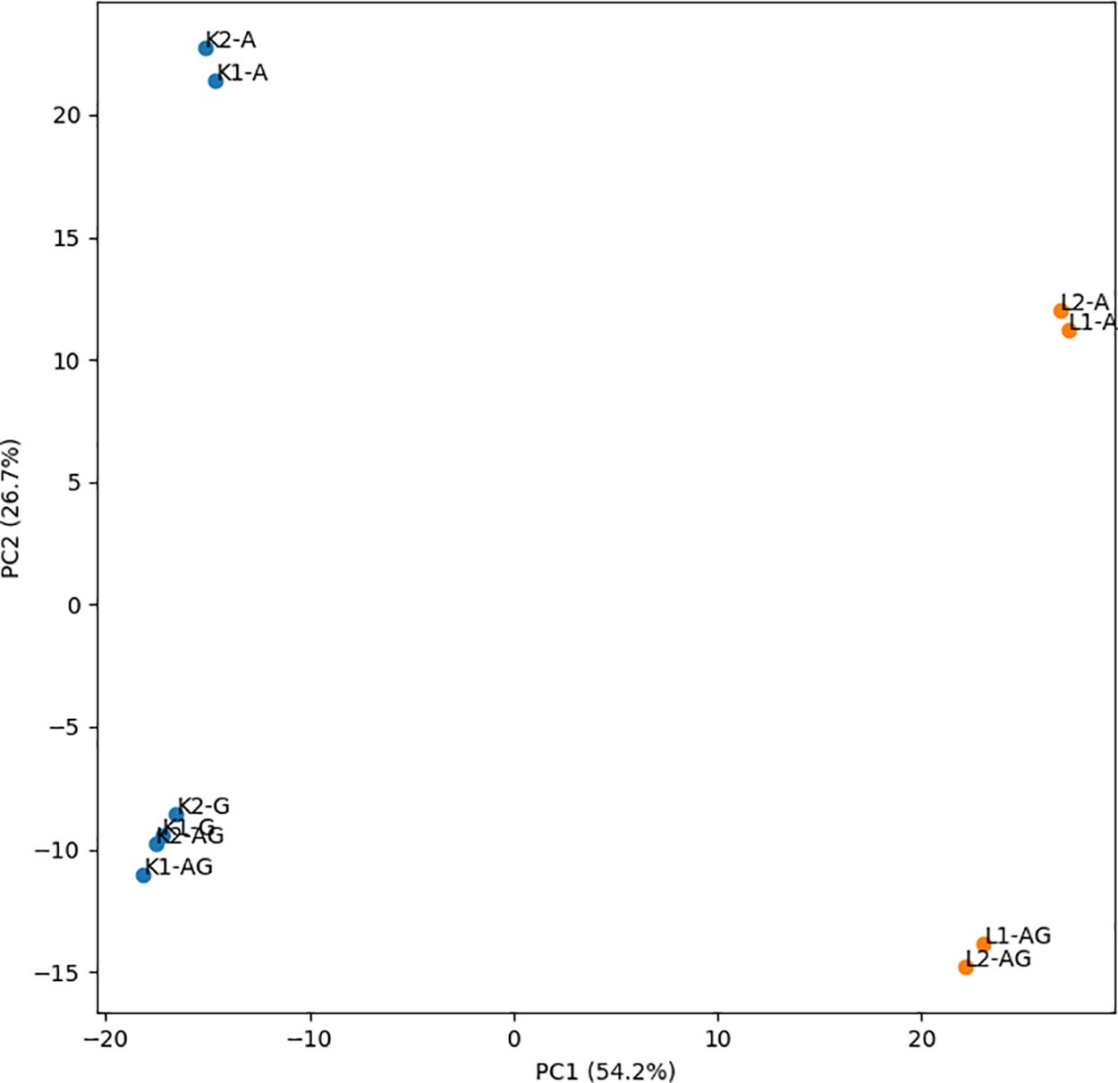
Principal component analysis of immune repertoires in different 5’-RACE libraries of individual L (orange) and K (blue) based on V-J composition.

We next examined whether an additional gel extraction affected the immune repertoire, which was measured by the frequencies of all possible V-J combinations. The distances between immune repertoires of 5’-RACE libraries were visualized using principal component analysis. Fig 6 confirms the high reproducibility between technical repeats for all protocols. It also reveals a difference in V-J composition between protocols A and AG. Because a library prepared by protocol A contained more short DNA fragments than one by protocol AG, this implies that V-J composition of the short DNA fragments differed from that of the long ones. In Fig 6, we also observed a high similarity between protocols AG and G. This indicates that the AMPure step did not affect the composition of the long DNA fragments.

Although a difference between libraries prepared by protocols A and AG was observed, the libraries were separated mainly along the second principal axis, which captured only 26.7% of the variance. In contrast, the libraries of the two individuals L and K differed mainly along the first principal axis, which represented more than half of the variation. In other words, the between-protocol variation was smaller than the between-individual variation. To develop a better idea about the variations in immune repertoire, we calculated Pearson correlations in V-J composition between the 5’-RACE libraries. The correlations between libraries of the same individuals were relatively high (e.g., 0.98 between L1-A and L2-AG) compared to those of different individuals (e.g., 0.60 between L1-A and K1-A) (S1 Table).

### Amount of data for studying immune repertoire

**Fig 7:**
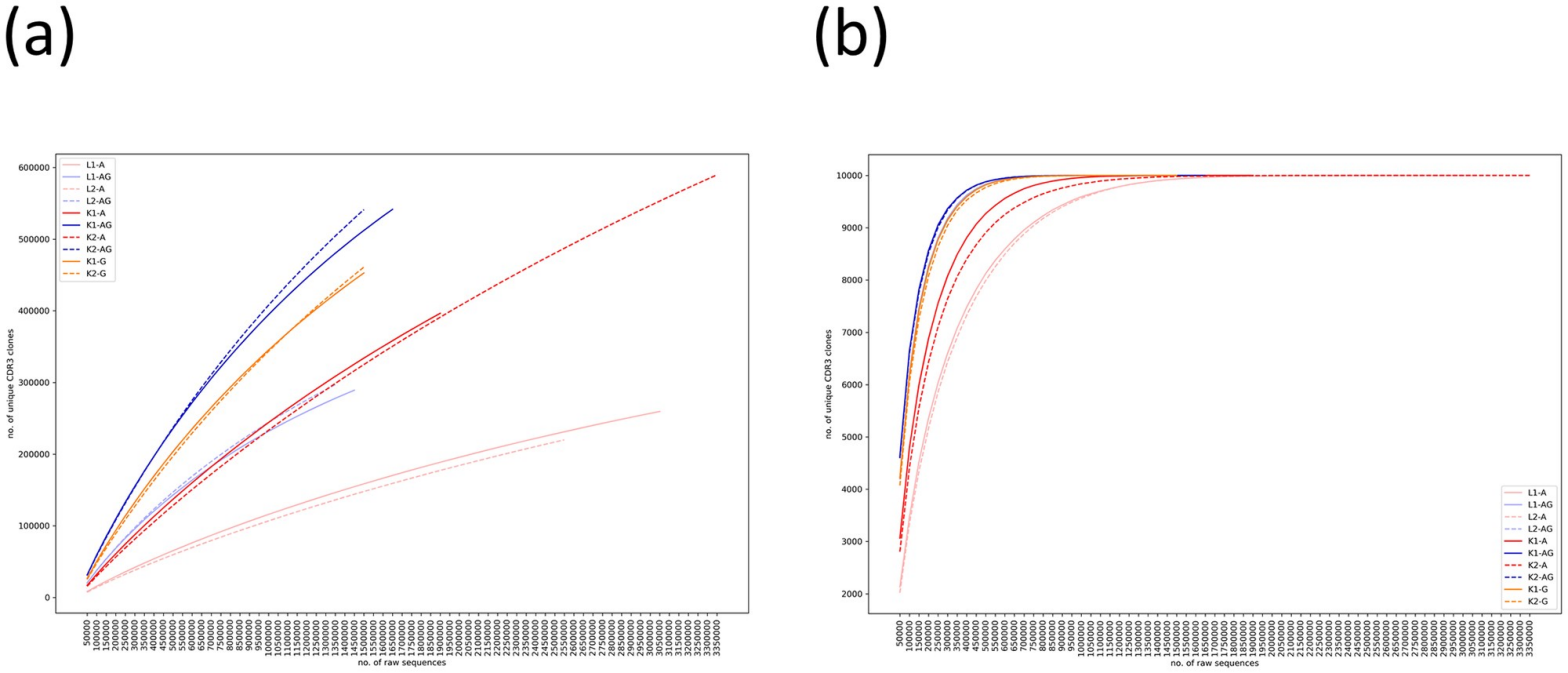
Rarefaction curves of (a) all and (b) top 10,000 unique CDR3 clones.

For studying immune repertoire, a practical issue is determining the amount of data required for a comprehensive exploration. For exploring immune repertoire, protocol AG should be more efficient than protocol A as it yielded a higher fraction of regular data. To quantify the efficiency, we counted the numbers of unique CDR3 clones captured by various amounts of raw PE reads. Fig 7a confirms that protocol AG discovered more unique CDR3 clones than protocol A with the same amount of raw data. As the curves had not plateaued, more reads were required to cover a majority of the unique CDR3 clones. Based on the Chao1 estimates, current amounts of raw reads (1.3–3.4 million reads) only covered about 41.7–61.3% of the estimated numbers of unique CDR3 clones in the 5’-RACE libraries (Table 2). For immune repertoire studies that focus more on the abundant CDR3 clones, the current amount of raw data was sufficient, e.g., to cover the top 10,000 abundant clones (Fig 7b). Using protocol AG, almost all the top 10,000 abundant clones could be found with one million raw PE reads.

**Table 2:** Statistics of CDR3 clones. The last column is the percentage of Chao1 estimates covered by the unique CDR3 clones.

Library	No. of CDR3 clones	No. of unique CDR3 clones	Chao1 estimates	Estimated % coverage of unique CDR3 clones
L1-A	879948	261427	493668	53.0
L2-A	659607	220222	528553	41.7
L1-AG	1149563	290417	473843	61.3
L2-AG	1081868	300764	558294	53.9
K1-A	833107	403731	909139	44.4
K2-A	1305756	591873	1226989	48.2
K1-AG	1403029	546357	902410	60.5
K2-AG	1264172	552352	1091811	50.6
K1-G	1077133	457743	841249	54.4
K2-G	999473	462991	1013819	45.7

## Discussion

To investigate factors behind the varying efficiency of 5’-RACE data, we explored the protocol of a commercial kit for preparing 5’-RACE library of TCR-β genes in this work. A commercial kit was selected because of its expected popularity. For example, the SMARTer kit had been used in several studies [8–10, 15–17]. Although various 5’-RACE protocols exist, amplification based on template-switching [18], like one for the commercial kit, is becoming a gold standard for TCR studies [6, 19]. Therefore, most current 5’-RACE protocols for preparing a TCR library follow a similar principle, which suggests that our findings could be extended in general.

Although this work focused on TCR-β, we did examine TCR-α sequencing for the control RNA sample at the beginning of the project. Using the commercial 5’ RACE protocol, about 86% of the data were regular TCR-α sequences. Therefore, efficiency is likely not an issue for TCR-α sequencing in a 5’ RACE approach. A possibility for the higher efficiency of TCR-α sequencing is less frequent non-regular recombination. As TCR-α does not contain a D gene segment, only one recombination step is needed. Compared to the two-step recombination of TCR-β, the chance for non-regular recombination may be lower. This conjecture requires further study, which is outside the scope of this work.

In the 5’-RACE data of the control sample, we observed that non-regular TCR-β sequences tended to be shorter than the regular ones. This led to our key discovery of a strong linear correlation between the fraction of short DNA fragments in the library and the fraction of non-regular TCR-β sequences in the data. The strong correlation indicates that the efficiency of 5’-RACE data is mainly controlled by the presence of short DNA fragments in the library. Therefore, we recommend conducting a fragment length analysis on the 5’-RACE products before the costly sequencing. The measured fraction of short DNA fragments can then be used to estimate the fraction of regular TCR sequences via the linear equation. If the estimated fraction is not satisfying, one may consider re-running the filtering step for an acceptable yield of regular data. The fraction of short DNA fragments is also a convenient metric for optimizing experimental conditions. In other words, our key discovery helps setting up a quality-control system to ensure high efficiency of 5’-RACE data. As deep sequencing of TCR genes is still relatively expensive, good quality control is valuable.

The linear regression in Fig 4 reveals that a 1% increase of short DNA fragments led to an ~4% increase of non-regular TCRβ sequences. For example, a 10% short DNA fragments corresponds to an ~44% non-regular TCR-β sequences. This is consistent with the known fact that shorter DNA amplifies more efficiently in NGS [20]. It also emphasizes the importance of effective filtering of short DNA fragments. Note that the linear regression has a positive intercept at the y-axis. This suggests that even complete removal of short DNA fragments still cannot achieve a 100% regular reads because some non-regular DNA fragments are still long. Indeed, among merged reads of the K1-AG library, 5.7% were non-regular TCR-β sequences and about half of which were longer than 512 bp (two standard deviations below mean length of the regular sequences).

Our findings were consistent with previous TCR studies. For example, Ruggiero et al. tried a RACE protocol without any filtering and found that only 30–40% of the TCR-β sequences were regular [11]. Alachkar et al. applied the commercial kit used in this work to examine TCR-β repertoire for kidney transplant recipients [10]. They followed the manufacture’s 5’ RACE protocol, which was expected to select TCR-β amplicons of size 400–900 bp using AMPure. However, their fraction of regular TCR-β sequences was also only 30–40%, which suggested an ineffective size selection. Using the same commercial kit, Fang et al. modified the 5’-RACE protocol for Ion-Torrent sequencing and conducted an additional size selection at 500–700 bp using Pipping Prep, which is a gel-based method [8]. In their 5’-RACE data of a healthy donor, 81% were regular TCR-β sequences. Mamedov et al. also recommended size selection via gel extraction [6], however, they did not elaborate on the rationale at all. Interestingly, Inoue et al. applied a 5’-RACE protocol similar to the one used by Fang et al. for examining TCR-β repertoire in melanoma tissues and found a varying fraction (8–75%) of regular TCR-β sequences [9]. Although the authors also used Pipping Prep for the additional size selection, the size range was not clear. We suspect that a size range 300–950 bp [15] instead of 500–700 bp was applied in that study. If that is the case, a good fraction of non-regular TCR-β sequences longer than 300 bp were still expected based on Fig 1. Consistent with our findings, these studies support that an accurate and effective size selection of TCR amplicons is crucial for data efficiency and should be implemented carefully.

A majority of the long non-regular TCR-β sequences showed a stretch that covered two neighboring J gene segments (e.g., J2-3~J2-4). Among those, the type J2-2P-J2-3 was particularly abundant. Most of the remaining long non-regular TCRβ sequences showed recombination of D and J gene segments, including intron at the immediate upstream of the D gene segment. As most of the long non-regular TCRβ sequences did not exceed 500 bp, the lower size limit should be at least 500 bp. Note that one needs to include the length of adapters for accurate gel extraction. The use of a different size range also implies that the source of non-regular TCR sequences or their length distributions is not well known by the science community, which can be better informed via our work.

Although a high fraction of regular data is usually desired for studying immune repertoire, non-regular TCR sequences may also be useful as they could be associated with disease status [8, 21]. For example, Fang et al. found that the fraction of non-regular TCR-β sequences in the 5’-RACE data of NSCLC patients (81.2%) was much higher compared to the healthy donor (13.8%). The authors were careful about the 5’-RACE protocol as they did an additional gel extraction to remove short DNA fragments. If no gel extraction is conducted, we recommend analyzing only long TCR sequences (e.g., >500 bp) to reduce noise stemming from the possible varying efficiency of filtering short DNA fragments by AMPure. On the contrary, if non-regular TCR sequences are desired, one may consider skipping the filtering step in the 5’-RACE protocol. In any case, the size control of RACE products is important in TCR analysis.

## Supporting information

S1 Table Pearson correlation of V-J compositions between 5’-RACE libraries. (DOCX)

S1 Fig Distribution of gap sizes between paired reads of unmergeable PEs of the control RNA sample. Gap sizes are calculated via subtracting the aligned positions of the last bases of read 1 and 2. In case an intron may exist within the gap, the intron length is subtracted.
(DOCX)

S2 Fig Gel extraction for the two repeats of 5’-RACE libraries (L1 and L2) constructed using protocol AG. (DOCX)

S3 Fig Gel extraction for the two repeats of 5’-RACE libraries (K1 and K2) constructed using protocol AG and G. (DOCX)

S4 Fig Integrity of three RNA samples. (DOCX)
