# Supplementary material for: Dissecting efficiency of a 5’ rapid amplification of cDNA ends (5’-RACE) approach for profiling T-cell receptor beta repertoire
Source: PLoS One. 2020 Jul 23;15(7):e0236366. doi: 10.1371/journal.pone.0236366 (PMC7377388; doi:10.1371/journal.pone.0236366)
Supplement: S1 Table — (DOCX) [file pone.0236366.s001.docx]

S1 Table. Pearson correlation of V-J compositions between 5’-RACE libraries.

|  | L1-A | L1-AG | L2-A | L2-AG | K1-A | K1-G | K1-AG | K2-A | K2-G | K2-AG |
| --- | --- | --- | --- | --- | --- | --- | --- | --- | --- | --- |
| L1-A | 1 | 0.986 | 0.999 | 0.979 | 0.598 | 0.592 | 0.569 | 0.605 | 0.597 | 0.577 |
| L1-AG | 0.986 | 1 | 0.984 | 0.998 | 0.576 | 0.591 | 0.569 | 0.585 | 0.594 | 0.576 |
| L2-A | 0.999 | 0.984 | 1 | 0.978 | 0.607 | 0.598 | 0.575 | 0.612 | 0.603 | 0.583 |
| L2-AG | 0.979 | 0.998 | 0.978 | 1 | 0.567 | 0.584 | 0.564 | 0.576 | 0.586 | 0.571 |
| K1-A | 0.598 | 0.576 | 0.607 | 0.567 | 1 | 0.980 | 0.977 | 0.997 | 0.981 | 0.979 |
| K1-G | 0.592 | 0.591 | 0.598 | 0.584 | 0.980 | 1 | 0.996 | 0.982 | 0.999 | 0.998 |
| K1-AG | 0.569 | 0.569 | 0.575 | 0.564 | 0.977 | 0.996 | 1 | 0.976 | 0.993 | 0.999 |
| K2-A | 0.605 | 0.585 | 0.612 | 0.576 | 0.997 | 0.982 | 0.976 | 1 | 0.984 | 0.980 |
| K2-G | 0.597 | 0.594 | 0.603 | 0.586 | 0.981 | 0.999 | 0.993 | 0.984 | 1 | 0.996 |
| K2-AG | 0.577 | 0.576 | 0.583 | 0.571 | 0.979 | 0.998 | 0.999 | 0.980 | 0.996 | 1 |
