# Supplementary material for: Dissecting efficiency of a 5’ rapid amplification of cDNA ends (5’-RACE) approach for profiling T-cell receptor beta repertoire
Source: PLoS One. 2020 Jul 23;15(7):e0236366. doi: 10.1371/journal.pone.0236366 (PMC7377388; doi:10.1371/journal.pone.0236366)
Supplement: S1 Fig — Gap sizes are calculated via subtracting the aligned positions of the last bases of read 1 and 2. In case an intron may exist within the gap, the intron length is subtracted. (DOCX) [file pone.0236366.s002.docx]

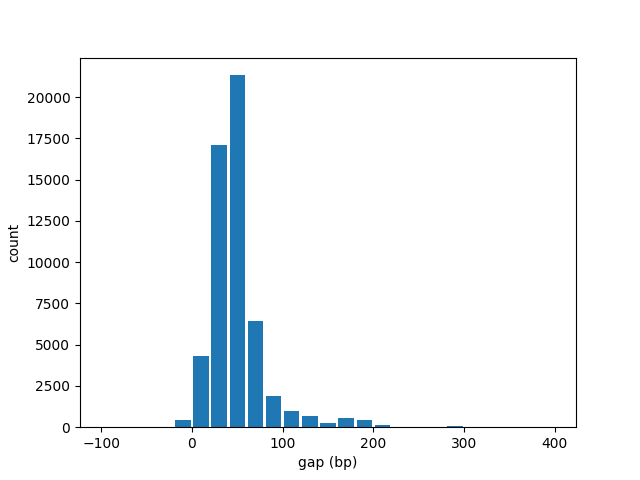


S1 Fig. Distribution of gap sizes between paired reads of unmergeable PEs of the control RNA sample. Gap sizes are calculated via subtracting the aligned positions of the last bases of read 1 and 2. In case an intron may exist within the gap, the intron length is subtracted.
