# Supplementary figures and images for: Dissecting efficiency of a 5’ rapid amplification of cDNA ends (5’-RACE) approach for profiling T-cell receptor beta repertoire
Source: PLoS One. 2020 Jul 23;15(7):e0236366. doi: 10.1371/journal.pone.0236366 (PMC7377388; doi:10.1371/journal.pone.0236366)

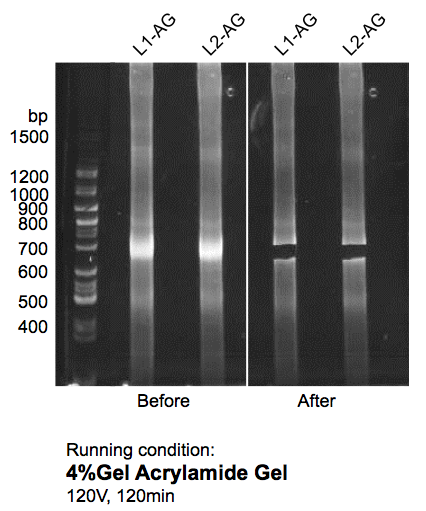


S2 Fig. Gel extraction for the two repeats of 5’-RACE libraries (L1 and L2) constructed using protocol AG.

Supplement: S2 Fig — (DOCX) [file pone.0236366.s003.docx]

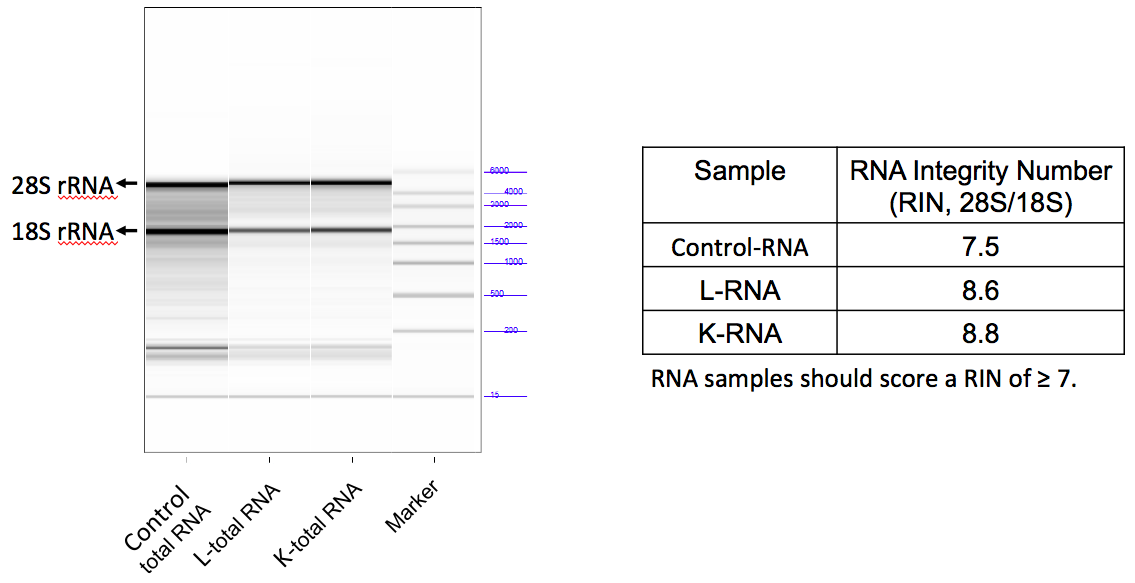


S4 Fig. Integrity of three RNA samples.

Supplement: S4 Fig — (DOCX) [file pone.0236366.s005.docx]
